# Mobility and strength training with and without protein supplements for pre-frail or frail older adults with low protein intake: the Maximising Mobility and Strength Training (MMoST) feasibility randomised controlled trial protocol

**DOI:** 10.3310/nihropenres.13507.1

**Published:** 2023-11-24

**Authors:** Kavita Biggin, Ioana R. Marian, Sarah E. Lamb, Alana Morris, Caoileann Murphy, Andrew Carver, Nirvana Croft, Esther Williamson

**Affiliations:** 1 Nuffield Department of Rheumatology, Orthopaedics and Musculoskeletal Sciences, The Botnar Research Centre, University of Oxford, Oxford, England, UK; 2 Oxford Clinical Trials Research Unit, Centre for Statistics in Medicine, Nuffield Department of Orthopaedics, Rheumatology and Musculoskeletal Sciences, The Botnar Research Centre, University of Oxford, Oxford, England, UK; 3 Faculty of Health and Life Science, University of Exeter, Exeter, England, UK; 4 Australian Catholic University, Fitzroy, Victoria, Australia; 5 Teagasc Food Research Centre, Ashtown, Dublin, Ireland; 6 Patient and Public Involvement Representative, University of Oxford, Oxford, England, UK

**Keywords:** frail, pre-frail, protein supplements, mobility, strength

## Abstract

**Background:**

Frailty is a common syndrome affecting older people and puts them at risk of hospitalisation, needing care or death. First signs of frailty include reduced muscle strength and mobility decline. A key cause of mobility decline as we age is sarcopenia (age related reduction in muscle strength and mass). Poor nutrition contributes to sarcopenia. A shortfall in protein is associated with reduced muscle mass and strength. This may be due to inadequate intake but also because older people have higher protein needs, especially those with multimorbidity. We need to develop effective treatment to reduce or slow the onset of frailty and mobility decline. Exercise is a recommended treatment. Protein supplements to address the shortfall in protein have the potential to enhance the benefit of regular exercise in frail or pre-frail older adults. This has yet to be definitively demonstrated.

**Aim:**

To establish the feasibility of conducting an RCT evaluating mobility and strength training with or without protein supplements for people over 60 years old who are frail or pre-frail with a low protein intake.

**Methods:**

A multicentre, parallel, 2-group, feasibility RCT. Participants (recruitment target = 50) with problems walking, low protein intake and classified as frail or pre-frail will be recruited from four NHS Physiotherapy community services. Participants will be randomised (secure computer-generated: 1:1) to receive 24 weeks of mobility and strength training (delivered in 16 group sessions plus home exercises) or 24 weeks of mobility and strength training with daily protein supplements. Primary feasibility objectives are to estimate 1) ability to screen and recruit eligible participants, 2) intervention fidelity, adherence, and tolerance and 3) retention of participants at follow up. Secondary objectives are to 1) test data collection procedures, 2) assess data completeness and 3) confirm sample size calculation for a definitive RCT.

**Registration:**

ISRCTN Registry ( ISRCTN30405954; 18/10/2022).

## Plain language summary

As people get older, they may become frail and become less able to deal with illness or injury. People with frailty are more likely to fall or need care. We need to find ways to stop people becoming frail or slow the progress of frailty so older people can live independently.

Exercise is a treatment for frailty that can improve muscle strength and walking. Including extra protein in an older person’s diet may also help. Protein provides the building blocks for muscles, but many older people do not eat enough protein. Taking extra protein while exercising may increase the benefits of exercise, but we do not know if it reduces frailty or improves walking and quality of life.

We want to conduct a large clinical trial comparing mobility and strength training plus extra protein to training alone. This study aims to test if it is possible to carry out a large trial by finding out:

•   Can we recruit enough participants with low protein intake?

•   Do participants attend the exercise classes and take the protein supplements?

•   Do participants attend follow-up assessments?

We aim to recruit up to 50 older people who are frail/at risk of frailty and have low protein intake. We will identify participants *via* NHS Community Trusts, from an existing cohort study and by advertising in the community. Participants are randomly allocated to training plus protein or training only. Training involves muscle strengthening, balance, and walking exercises. Participants will attend a weekly exercise class with a physiotherapist for 16 weeks and do exercises at home once/week. They are then asked to exercise at home twice weekly for a further 8 weeks. Half the participants will take extra protein while exercising.

At enrolment and 8 months later, we collect information on frailty, walking ability, muscle strength and quality of life.

## Introduction

Frailty is a state of vulnerability to adverse outcomes, including death, hospitalisation and dependence that is not accounted for by known diseases experienced by many people as they get older ^1^. Muscle strength, muscle mass, and mobility are central to the concept of frailty. The first manifestations include declines in muscle strength and mass, and changes in mobility such as reduced walking speed ^2^. These features of frailty are potential treatment targets as we look to develop ways to prevent or slow the onset of frailty in older people to help them maintain their independence and quality of life and reduce health and social care needs. Mobility limitations in older age are primarily due to progressive declines in muscle strength and mass caused by the effects of age on muscle known as sarcopenia. Inactivity also contributes to this decline. The maintenance of good mobility in older age is intrinsically linked to maintaining muscle strength and mass.

Poor nutrition is also thought to contribute to sarcopenia and, therefore, reduced mobility and frailty. A shortfall in protein can lead to loss of muscle mass ^3^. The European Society for Clinical Nutrition and Metabolism (ESPEN) recommend that older adults should consume 1-1.2 g protein/kg body weight (BW)/day to support the maintenance of muscle mass and function ^4^. Furthermore, ESPEN recommend higher protein intakes of 1.2-1.5 g/kg BW/day in those with chronic disease such as frailty ^4^. It is estimated that approximately half of older adults do not achieve 1.0 g protein/kg BW/day ^5^.

Exercise including progressive resistance and balance training is a core strategy recommended for addressing reduced muscle strength and mass and mobility decline, which contribute to frailty ^6, 7^. However, there is uncertainty as to whether protein supplements are an effective intervention to reverse or slow the onset of frailty or mobility decline. The findings of randomised trials are mixed, and complicated by different settings, samples, formulations, timing, duration and dose ^8^. Current evidence suggests that nutritional interventions such as increasing protein intake on their own do not improve frailty status or frailty associated physical performance measures such as mobility in community dwelling older adults ^9– 11^. International guidelines recommend that protein supplementation should only be considered when undernutrition is diagnosed ( *e.g.*, insufficient dietary protein) and supplements should be offered in conjunction with exercise ^8^. These recommendations are based on low and very low certainty of evidence. Many studies to date have not specifically recruited participants with low protein intake, which may dilute the effect of supplementation on outcomes, nor have they tailored the protein supplementation to individual participants and many only report short term outcomes. There is a need for trials evaluating long term outcomes including cost-effectiveness and outcomes such as quality of life, which are rarely reported ^7^.

We have developed and tested a rehabilitation programme that focuses on muscle strength and mobility training ^12^, which resulted in clinically significant increases in physical performance ^13^. It is possible that these benefits could be enhanced by also optimising poor dietary protein intake. We have designed a randomised controlled trial to test this hypothesis, and which will address the shortcomings of previous trials including targeting a population most likely to benefit from protein supplementation (those with low protein intake), tailoring protein provision, and collecting outcomes needed to make clinical recommendations such as quality of life and health and social care use to estimate cost-effectiveness. However, a fully powered trial would be large and complex and, therefore, a feasibility trial is needed to establish if we can successfully undertake this randomised control trial (RCT).

### Aim

The aim of this study is to establish the feasibility of conducting an RCT to evaluate the clinical and cost-effectiveness of mobility and strength training plus protein supplements for pre-frail and frail community dwelling older adults with low protein intake compared to training alone.

## Methods

### Trial design

**Figure 1.**
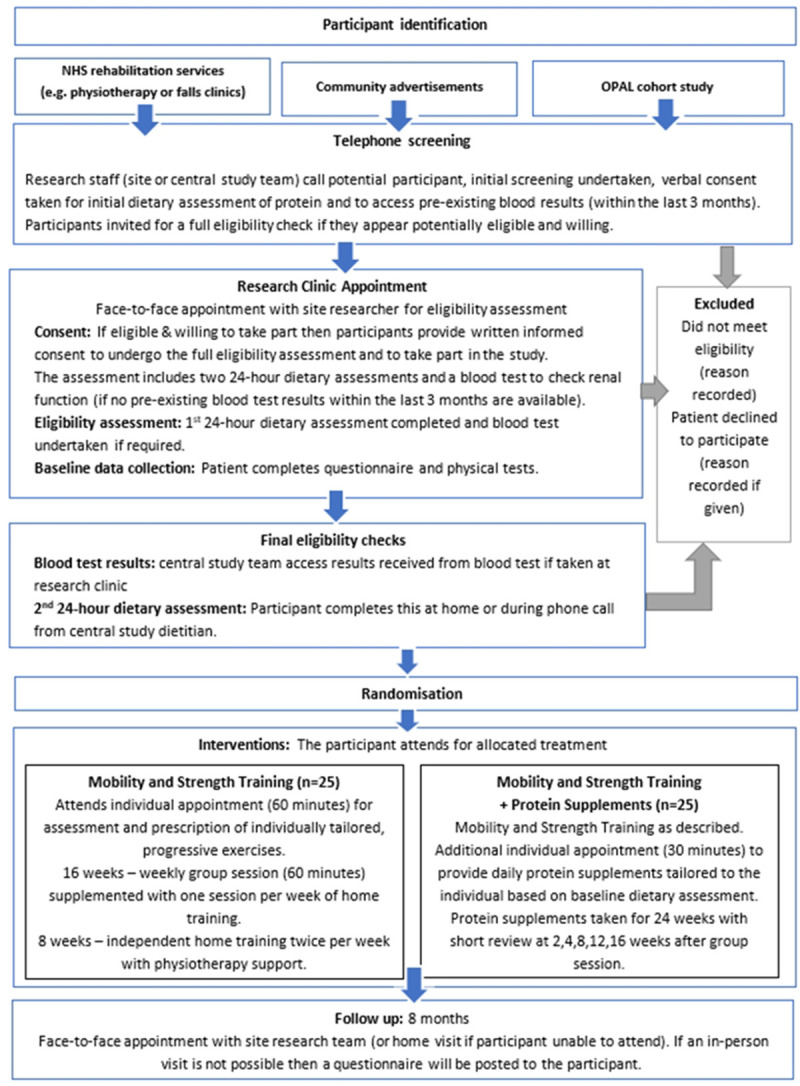
Study flow chart.

A muti-centred, parallel, 2-group, feasibility randomised (1:1) controlled trial. A study flow chart is presented in Figure 1. This protocol has been reported following the Standard Protocol Items: Recommendations for Interventional Trials (SPIRIT) statement. MMoST Patient Information Sheet and Consent Form can be found as *Extended data* ^14^.

### Setting

Participants will be identified from three sources:

1. NHS community trusts.

2. From an existing primary care-based cohort study.

3. Community advertising.

Interventions will be delivered within community physiotherapy services at four NHS sites across England.

### Eligibility criteria

The target population is adults aged 60 years and older who are either frail or pre-frail (as defined by the Fried Frailty Criteria), reporting walking difficulties/slow walking, and low protein intake (<1 g protein/kg BW/day). The full eligibility criteria are described in Table 1.

**Table 1.** Eligibility criteria.

Inclusion criteria
Aged 60 years and older. Frail (at least 3 criteria) or pre-frail (1-2 criteria) as defined by the Fried Frailty criteria ^16^. This must include slow walking speed (or difficulty walking). Low protein intake (<1 g protein/kg body weight/day) measured by the average of two 24-hour dietary recall assessments. Willing and able to provide informed consent to participate.
Exclusion criteria
Dementia or cognitive impairment (defined as an Abbreviated Mental Test (AMT) score of 6 or less). Inability to walk 3 m without assistance (walking aid permitted). Unable to follow verbal instructions that would make participation in the exercise group impractical including severe hearing impairment not corrected by a hearing aid or inability to follow simple safety instructions ( *e.g.*, English comprehension). Living in a residential care or nursing home. Pre-existing diagnosis of: ▪ Stroke in the last 6 months. ▪ Parkinson’s Disease. ▪ Acute, unstable physical illness that would make participation in the exercise programme unsafe. ▪ Dysphagia or swallowing problems that requires a modified diet. ▪ Type 1 diabetes or Type 2 diabetes on insulin. ▪ Severe kidney disease (stage 4 or 5). Already taking protein supplements or known allergies to ingredients of protein supplement (milk, soya) or lactose intolerant. Poor kidney function defined by an Estimated Glomerular Filtration Rate (eGFR) of <30 mL/min/1.73m2 (blood test). High risk of developing refeeding problems based upon items from NICE guidance ( https://www.nice.org.uk/guidance/cg32).

### Participant identification

Clinicians such as physiotherapists working in the community trusts will identify potential participants from their current caseload or from waiting lists. They then provide patients with the Participant Information Leaflet (PIL) and seek verbal permission to pass their contact details to the research staff for screening.

We have ethical approval to recruit *via* an existing cohort study (The Oxford Pain, Activity and Lifestyle (OPAL) study ^15^. The OPAL study team will send out invitation letters to potentially eligible OPAL participants (based on response from their most recent questionnaire) living near to study sites and have given permission to be contacted about opportunities to take part in clinical trials. They also send a copy of the PIL and a response form to return to the study team. Interested OPAL participants are contacted by research staff for an eligibility assessment.

We will advertise the study in community venues used by older people that are close to the study sites including retirement living centres and community centres. Posters have a QR code so that interested people can register their interest and give permission to be contacted by the study team or they can telephone the study team.

### Screening

***Telephone screening.*** Initial telephone screening will be undertaken by site research staff. Assessment of frailty is based on self-report by the potential participants using the Fried Frailty Criteria (unintentional weight loss [6 kg or more during the last six months, or 3 kg or more during the last month]; experienced weakness or poor handgrip strength; self-reported exhaustion; slow walking speed (or difficulty walking); and low physical activity) ^16^. Frail is defined by the report of at least three criteria and pre-frail by the report of 1-2 criteria. Participants must report slow walking speed (or difficulty walking).

As this initial screening is done over the telephone, verbal consent will be gained to undertake a short dietary questionnaire ( https://proteinscreener.nl) ^17^ to assess if protein intake is likely to be low. Verbal consent to undertake this screening will be confirmed and then recorded on the REDCap database before proceeding. Verbal consent was deemed adequate by the Research Ethics Committee. Participants who score at least a 30% risk of low protein intake (<1g/kgBW/day) are eligible to undergo further assessment. We will seek verbal permission to view any blood test results taken within the last three months to check renal function. Potential participants who appear eligible after completing telephone screening will be invited to attend a research clinic assessment at their local site. Potentially eligible participants are asked to complete a 24-hour food diary the day prior to their appointment to aid the dietary assessment to estimate protein intake.

***Informed consent, trial specific screening procedures and baseline data collection.*** Participants will attend a face-to-face research clinic appointment with a researcher. The researcher will provide verbal information about the study and the participants will be given the opportunity to ask questions before being asked to provide written informed consent to undertake the final elements of screening and to take part in the trial. Following consent, cognitive screening using the Abbreviated Mental Health Test (AMT) ^18^ and assessment of protein intake will be completed as well as taking a blood test to check renal function if required. We will obtain permission from the participants to inform their GP of their participation. Researchers responsible for consenting participants will be trained in consent procedures and been delegated to do so by the site Principal Investigator.

Estimated daily protein intake will be established by taking the average of two 24-hour dietary assessments using an online dietary assessment tool (myfood24.org) ^19^. The first dietary analysis is completed during this appointment. If protein intake is >1.4 g protein/kgBW/day for the first assessment, then they will be excluded at this point as it is unlikely their second dietary assessment will result in an average intake of <1.0 g protein/kgBW/day. The second dietary assessment will be completed after the appointment, which potential participants can complete online or with help from the central study team dietitian over the telephone.

If the participant has not had a blood test in the last three months to check their renal function, then a capillary blood sample will be taken to test Estimated Glomerular Filtration Rate (eGFR) using test kits provided Medichecks.com Ltd. The participant will be asked to complete a baseline questionnaire and undergo physical tests ( Table 2).

**Table 2.** Data collection.

Domain	Measure	Baseline	Weeks 1–16	Weeks 17–24	8 month follow up
Demographic	Date of birth, sex, ethnicity, relationship status, height and weight * (body mass index), index of deprivation (postcode), type of housing, work status, education, carer needs, household income.	x			Height, weight only
Physical capacity	Short Physical Performance Battery (SPPB) ^20^ (proposed primary outcome)				
Mobility	6-minute Walk Test * ^21^	x			x
Muscle strength and sarcopenia	Hand grip * ^22^ and quadriceps strength * ^23^ measured using dynamometer SARC-F Questionnaire ^24^	x			x
Frailty	Tilburg Frailty Indicator (TFI) ^25^ Clinical Frailty Scale Health Questionnaire to calculate the Clinical Frailty Scale Score) ^26^ Fried Frailty Index based on self-report ^27^	x			x
Falls	Prevention of Falls Network Europe (ProFANE) self-report of falls and injuries ^28^	x			x
Comorbidities	Nordic pain questionnaire ^24, 25, 29^; self-reported health conditions ^30^	x			x
Protein intake	Assessment of daily dietary protein intake (myfood24.org)	x			x
Renal function	Blood test to evaluate Estimated Glomerular Filtration Rate (eGFR)	x			x
Quality of life	EuroQol Group 5-Dimension Questionnaire (EQ-5D-5L) ^31^	x			x
Intervention data	Intervention logs; protein supplement review logs (physio and/or dietitian completed) Home diaries: exercise and protein supplements		x	x	
Health Resource Use	Client Service Receipt Inventory ^32^				x

*Physical tests conducted by researcher

On completion of this appointment, participants will be advised that they may be excluded from the study depending on their blood test results or second dietary assessment.

***Confirmation of eligibility assessment.*** Final eligibility checks will be completed by the central study team prior to randomisation including blood test results, second dietary assessment and whether they risk of refeeding. The central study team dietitian will assess for risk of refeeding using information from the dietary assessment. The central study team will phone the participant if further clarification is needed prior to final confirmation of eligibility.

### Randomisation, blinding and allocation concealment

Eligible participants will be randomised to Mobility and Strength Training or Mobility and Strength Training with protein supplements using a secure (encrypted) centralised web-based randomisation service provided by the Oxford Clinical Trials Unit (OCTRU). Randomisation will be 1:1, stratified by frailty (pre-frail/frail) using variable block sizes within each strata. Following randomisation, the physiotherapy team and the central study team will be notified of allocation *via* automated email. As we are not using a placebo supplement, it is not possible to blind the participants or physiotherapists delivering the interventions (including provision of supplements). Outcome assessors will be blinded to treatment allocation.

### Interventions

The schedule of intervention delivery is outlined in Table 3.

**Table 3.** Intervention delivery schedule.

	Mobility and Strength Training	Training + Protein Supplements
Individual pre-class appointment		
Assessment by physiotherapist to establish current ability and exercise setting. Home exercise diary introduced.	X	x
Assessment by physiotherapist to discuss protein supplement dosing and instructions. Provided with written instructions and introduced to protein supplement diary.		x
Delivery of protein supplement arranged.		x
Weeks 1–16		
Weekly group mobility and strength exercises • Group discussion including behavioural strategies to encourage adherence with home exercises (goal setting, exercise planning and problem solving) (15 minutes) • Seated warm up (5 minutes) • Exercise circuit (20 minutes): Strengthening exercises: knee extension, hip extension and abduction, sit to stand Combined hip flexor and calf stretch Balance exercise • Walking circuit (20 minutes) One session of home exercises each week (exercise diary completed). Weights for home exercises are provided if appropriate.	x	x
Protein supplements taken daily. Complete supplement diary.		x
Protein Supplement Reviews including weight checks undertaken at weeks 2, 4, 8, 12 and 16 of the programme. Referral to dietitian if needed.		x
Weeks 17–24		
Twice weekly home exercises. Complete exercise diary.	x	x
3 follow up telephone calls to review progress with home exercises conducted by physiotherapist.	X	x
Protein supplements taken daily. Complete supplement diary.		x
3 telephone supplement reviews during the follow up telephone calls. Weight is not to be monitored.		x

***Mobility and Strength Training.*** All participants will be asked to undertake twice weekly mobility and strength training supported by a physiotherapist for 24 weeks using a combination of weekly group exercise (16 weeks) and home exercises (8 weeks). Group sessions are used to provide supervision and support to participants and promote adherence to long term exercise. This is a pragmatic approach to strength and mobility training that can be implemented within NHS physiotherapy departments without the need for specialist exercise equipment. We have delivered a similar programme for participants with mobility limitations due to spinal stenosis, which was well attended, the exercises were well tolerated and enjoyed by participants ^12, 33^.

The programme is progressive and tailored to each participant ( Table 3). Each participant will attend an individual appointment with the physiotherapist prior to attending the group. During this appointment, they undergo an assessment to evaluate their physical ability and are provided with their personal programme to be undertaken during the group sessions. The participants are provided with a workbook containing information about the exercises, exercises diaries and summaries of discussion topics.

Each group session follows the same format beginning with a short group discussion utilising behaviour changes strategies including goal setting and exercise planning to encourage adherence to the programme. Discussion topics include:

- The benefits of exercise and physical activity
- Building strength
- Dealing with aches and pain when exercising
- Goal setting and exercise planning
- Staying steady
- Making exercise and physical activity part of your life
- Digital options to support long term exercise
- Celebrate success

Seated warm up exercises are performed. Participants will then undertake an exercise circuit consisting of strengthening exercises focusing on the muscles required for safe walking and uses weights or resistance bands to make the exercises sufficiently challenging. The Borg Rating Scale of Perceived Exertion is used to allow individual tailoring while ensuring the exercise is sufficient to achieve gains in muscle strength ^34^. During the strengthening exercises, physiotherapist will ask participants to work at a level of 5-6/10 on the Borg Scale. Over the 16 weeks of group sessions, as muscles strengthen, exercises are progressed by manipulating the load and the number of sets and repetitions to ensure that participants continue to work at the expected level on the Borg scale. Load will be added using ankle weights, hand weights, weight vests or resistance bands. Speed is also added to introduce a power element to the strength training and a means of further progression. Finally, the exercise circuit includes a balance exercise that is progressed in difficulty and a lower limb stretch to ensure adequate mobility at the hip and ankle for walking.

The final element of the exercise programme is a supervised walking circuit. This includes exercises to challenge and improve walking balance and walking practice to increase speed and endurance. Less able participants may start off walking between two points with rest stops.

The exercises performed during the supervised group sessions are also undertaken at home. Participants are asked to complete the programme at home once a week while attending the group sessions. Then, twice per week for a further eight weeks of independent exercise (with three support phone calls). During group discussions and support phone calls, we help participants make a long-term exercise plan. This includes continuing with their home exercises but also considering other activities they may undertake include digital options. Participants will be given access to an existing exercise app (Good Boost: https://www.goodboost.ai/). Use of the app is completely optional and as part of this feasibility study, we are interested to see how many participants opt to use this type of offering.

***Protein supplements.*** Participants randomised to receive protein supplements will be asked to take the supplements for 24 weeks starting on the day they begin the group exercise sessions. The protein is a powdered supplement (Nutricia Fortifit) produced by Danone Nutricia Research and delivered to participants’ homes. Cold water is added to make up a drink in a shaker (125 ml water per serving). Each serving of the protein powder contains 149 kcal, 20.7 g whey protein, 2.8 g leucine, 20 ug/800 IU vitamin D and 501 mg of calcium. This product has been chosen as it is enriched with leucine, allowing more effective muscle protein synthesis stimulation in a lower serving ^35^, and is less calorific than using a pre-prepared protein drink. The main target of the intervention is to increase protein intake rather than energy.

Physiotherapists will provide the supplement to participants with the support of the central study team dietitian. Protein supplements are tailored to each participant based on their baseline protein intake. The aim is for each participant to consume up to 1.6 g protein/kgBW/day. This is slightly higher than the recommended daily protein intake for older adults with acute or chronic diseases to maintain or regain muscle mass (1.2 – 1.5 g protein/kgBW/day) ^4^. However, retrospective breakpoint analysis conducted on RCTs exploring the impact of protein supplementation on resistance training-induced gains in fat-free mass (FFM) demonstrated that increases in FFM plateaued at daily protein intakes of 1.6 g/kg/d ^36^. If a participant has a BMI of 30 or more, then the recommended dose of protein is 75% of the calculated amount based on weight (as per usual clinical practice) ^37^. Additionally, if a participant has an eGFR between 30-60 ml/min/1.73 m ^2 ^(indicating a moderate reduction in renal function), protein intake will be limited to 1.3 g/kgBW/day. This is the maximum dose recommended for this patient group by The Kidney Disease Improving Global Outcomes (KDIGO) guidelines.

On a day when participants undertake Mobility and Strength training (at the hospital or at home), they will be advised to take one of their drinks of the supplement directly after the exercise session. Participants will complete a protein supplement diary to monitor adherence and record any side-effects experienced. Participants will be monitored and weighed at regular reviews ( Table 3). If a participant is experiencing gastro-intestinal problems, then they will be advised to reduce the dose by a half and gradually increase the dose back to the recommended amount over four days to improve tolerance. If a participant has lost >10% of their weight or is reporting ongoing intolerance issues, then participants will be reviewed by the main study team dietitian to decide if the protein supplement dose needs to be adjusted or other dietary advice given.

The chosen supplement also contains vitamin D and calcium. If participants are already taking over the counter supplements, then they will be asked to stop taking them while they are on the protein supplements. If participants are already on prescribed supplements, then the physiotherapist will liaise with the participant’s general practitioner to adjust their prescription to ensure daily maximum doses are not exceeded (Vitamin D: maximum of 4,000 IU per day; Calcium: maximum of 1 g per day).

All participants will attend the same exercise sessions. Supplement reviews will be conducted outside these sessions and participants will be asked not to discuss them in the groups. Groups will be run this way to so sites can fill the exercise groups in a timely way.

### Training

Site research staff who are responsible for screening, consenting and data collection will attend approximately 6 hours of training to cover all aspects of the trial and processes involved. Physiotherapists delivering the interventions will attend a full day of training to learn how to deliver the group sessions, prescribe and monitor provision of protein supplements and to complete study related administration. All staff will receive a detailed study manual.

### Primary feasibility outcomes

***Progression criteria.*** Progression criteria into a future definitive trial will be based on the stop-go criteria assessing feasibility detailed in Table 4 alongside consideration of adverse events and discussions with the Independent Monitoring Committee. The stop-go criteria follow a ‘traffic light’ system: Red (stop), Amber (proceed with modifications) and Green (Go). There are four categories: recruitment, intervention fidelity, intervention adherence and study retention.

**Table 4.** Stop-Go Criteria.

	Red	Amber	Green
Recruitment: Number of participants recruited	< 25 participants recruited within 6 months	25–39 participants recruited within 6 months	>40 participants recruited within 6 months
Intervention fidelity: provision of the mobility and strength training	<50% undergo their individual assessment and are allocated to their group sessions	50% – 80% of participants undergo their individual assessment and are allocated to their group sessions	>80% of participants undergo their individual assessment and are allocated to their group sessions
Intervention fidelity: delivery of the mobility and strength training (fidelity assessment *via* observations based on core criteria checklist)	<50% of all key criteria are met during observed sessions	50–80% of key criteria are met during observed sessions	>80% of key criteria are met during observed sessions
Intervention fidelity: protein supplements	<50% of participants receive the protein supplements *via* home delivery as intended	50–80% of participants receive the protein supplements *via* home delivery as intended	>80% of participants receive the protein supplements *via* home delivery as intended
Intervention adherence: participant attendance at the mobility and strength training	On average, < 8/16 sessions (50%) of sessions attended by 70% or more participants	On average, 8-10/16 session (50–69%) of sessions attended by 70% or more participants	On average, at least 11/16 sessions (70%) attended by 70% or more of participants
Intervention adherence: participant adherence to the protein supplements	On average, 70% or more participants report taking protein supplements on <50% of days over 24 weeks	On average, 70% or more participants report taking protein supplements on 50–70% of days over 24 weeks	On average, 70% or more participants report taking protein supplements on ≥70% of days over 24 weeks
Study retention: follow up rates and checking for differential loss to follow up	>30% loss to follow up at 8 months in either study arm.	29–15% loss to follow up at 8 months in either study arm.	<15% loss to follow up at 8 months in both study arms.

Recruitment: We will collect data on screening and recruitment of eligible patients from each site on a monthly basis alongside reasons for exclusion.

Intervention fidelity: Physiotherapists will record intervention provision on an intervention log, which will be monitored to assess treatment fidelity. We will also observe or record intervention sessions and assess the delivery of the interventions using a checklist of the core criteria of the intervention. We will monitor protein supplement delivery to ensure supplements are delivered to participants as intended. The central study team will also visit up to 20% of participants at home to observe home exercises and check on protein supplement intake.

Adherence with the intervention: Attendance at the exercise classes is monitored using intervention logs completed by the physiotherapists. Participants will be asked to record when they take the protein supplements in their supplement diary.

Study retention: Follow up rates will be collected, and we will evaluate if there is any differential loss to follow up by study group.

### Secondary exploratory outcomes

We will collect a range of clinical outcomes at baseline and 8-month follow up to determine the viability of data collection, and to inform the sample size calculation for the future definitive RCT based on the proposed primary outcome for the trial (Short Physical Performance Battery). The clinical and participant reported outcomes are listed in Table 2.

### Adverse events

Safety reporting will begin from the time of randomisation to 8 month follow up. All study staff have been trained to report adverse events (AEs) related to the study interventions. Given the age of participants, there are foreseeable AEs that should only be reported if they occur during or within two hours of any study interventions. These include acute infections, medical instability ( *e.g.*, worsening of heart failure), vestibular disorders or stroke and fall-related injuries. We anticipate participants may experience delayed onset muscle soreness (<72 hours) upon starting a new exercise programme, which is only reported if they are more persistent or severe than expected. We would also expect that participants taking protein supplements may experience mild gastrointestinal complaints including diarrhoea, constipation, bloating and loss of appetite. Participants are made aware of these, and they should be short lived and managed by adjusting their protein supplement dose. If symptoms are more severe and longer lasting than what is expected, then they will be reportable.

A serious adverse event (SAE) is an untoward medical occurrence that results in death; is life-threatening; requires inpatient hospitalisation or prolongation of existing hospitalisation; results in persistent or significant disability/incapacity; consists of a congenital anomaly or birth defect. We will follow reporting procedures in accordance with Good Clinical Practice (GCP) to report any SAEs that are potentially related to trial procedures.

### Sample size

Given this is a feasibility study to inform the design of a definitive study, the sample size was determined from a recent simulation study of sample size requirements in pilot RCTs ^38^. A total of 50 measured subjects are recommended to calculate key design parameters (namely, standard deviation around the primary outcome measure for a definitive trial) and subsequently establish the definitive study sample size. Therefore, the target sample size for this study is 50 patients.

### Withdrawals of participants

During the trial, all participants have the right to withdraw at any time. In addition, site staff or the central team dietitian may discontinue a participant from a specific part of the intervention if they consider it necessary. Participants may withdraw from either protein supplements or exercise (but continue with the other part of the programme where applicable). They may also withdraw from the study including any further follow up assessments. Withdrawn participants will not be replaced.

### Data management

Site research staff enter the majority of the data directly into Research Electronic Data Capture (REDCap). Paper questionnaires are scanned and sent to the central study team. Physiotherapy staff involved in the intervention complete a paper intervention log, which is entered by the central study team into REDCap. Contact details are stored separately from the outcome data and will be deleted when no longer required as part of the study (within 12 months of the last data collection). All data is handled and stored in line with OCTRU Data Security procedures and Standard Operating Procedures which are in accordance with the Data Protection Act 2018, other relevant regulations and Good Clinical Practice (GCP) guidelines.

### Statistical methods

The primary analysis will evaluate the feasibility of conducting a future definitive multicentre RCT. Descriptive statistics of the following feasibility outcomes will be reported: recruitment rates and reasons for ineligibility, intervention fidelity and adherence, safety monitoring, retention rates, data completion rates. Data will be summarised and reported in accordance with Consolidated Standards of Reporting Trials (CONSORT) guidelines extension for feasibility studies ^39^. Baseline demographic and clinical characteristics for each group will be described. Participant reported outcomes will be summarised as a mean (standard deviations)/median (interquartile ranges) or frequency and percentage for continuous and categorical outcomes, respectively. We will explore missingness for each outcome to inform final selection of outcomes for the definitive RCT.

It is anticipated that all analysis will be undertaken using Stata (Stata Statistical Software: Release 16 or later, StataCorp LLC) or other well validated statistical packages. A trial statistician embedded within OCTRU will contribute to all statistical aspects of the study.

### Data monitoring

This study is coordinated by the United Kingdom (UK) Clinical Research Collaborative registered OCTRU at the University of Oxford. A rigorous programme of quality control has been implemented to ensure compliance to the current approved protocol, GCP, relevant regulations and OCTRU Standard Operating Procedures (SOPs). The Chief Investigator and the Trial Manager have developed data management and monitoring plans. The day-to-day management of the trial is the responsibility of the Chief Investigator, overseen by the Trial Management Group (TMG), who will meet monthly to assess progress. Quality assurance checks will be undertaken by the trial management team to ensure integrity of randomisation, study entry procedures and data collection. Additionally, the study may be monitored, or audited by sponsor or host sites in accordance with the current approved protocol, GCP, relevant regulations and standard operating procedures. We have established an independent monitoring committee to provide oversight of the study who meet regularly.

### Patient and Public Involvement

We worked with patient and public involvement (PPI) representatives to develop the funding application for this study, which included intervention and protocol development. Following the award of funding, a formal PPI group was established consisting of five members to assist with the conduct of the study. PPI representatives were all aged over 65 years old. The PPI group helped to refine the study protocol, develop study materials such as the patient information leaflet, test study procedures such as undertaking the protein assessment and developing the recruitment strategy. The study team meet with the PPI group 6 monthly to update them on progress and a member of the PPI group is a member of the TMG. There is a PPI representative on the Independent Monitoring Committee. On completion of this study, we will seek recommendations from our PPI group on the next steps for this research and work with them to produce a plain English summary for dissemination.

### Ethics and dissemination

This study was approved by the London-Surrey Research Ethics Committee, ref: 22/LO/0672.

Results will be published in a peer-reviewed journal with authorship eligibility according to the International Committee of Medical Journal Editors criteria. The final report will detail amendments to the study protocol. A plain language summary of the results will be posted to the trial participants.

## Trial status

The first site opened to recruitment on 01 March 2023. Recruitment is ongoing.

## Data Availability

No data are associated with this article. Figshare: MMoST Patient Information Sheet and Consent Form. https://doi.org/10.25446/oxford.24467452 ^14^. This project contains the following extended data: Patient Information Sheet Consent Form Data are available under the terms of the Creative Commons Attribution 4.0 International license (CC-BY 4.0).
