# Supplementary material for: Mobility and strength training with and without protein supplements for pre-frail or frail older adults with low protein intake: the Maximising Mobility and Strength Training (MMoST) feasibility randomised controlled trial protocol
Source: NIHR Open Res. 2024 Apr 8;3:62. Originally published 2023 Nov 24. [Version 2] doi: 10.3310/nihropenres.13507.2 (PMC11319903; doi:10.3310/nihropenres.13507.2)
Supplement: Peer review history

# Reviewer report: Reviewer response for version 2

**DOI:** 10.3310/nihropenres.14751.r31492

**Published:** 2024-05-08

**Authors:** Konstantinos Prokopidis

**Affiliations:** 1 Department of Musculoskeletal Ageing and Science, University of Liverpool, Liverpool, England, UK

I was able to see the comments and any changes the authors made. I approve this version of the manuscript.

Is the study design appropriate for the research question?

Yes

Is the rationale for, and objectives of, the study clearly described?

Yes

Are sufficient details of the methods provided to allow replication by others?

Yes

Are the datasets clearly presented in a useable and accessible format?

Not applicable

Reviewer Expertise:

Sarcopenia, frailty, malnutritio

I confirm that I have read this submission and believe that I have an appropriate level of expertise to confirm that it is of an acceptable scientific standard.

# Reviewer report: Reviewer response for version 1

**DOI:** 10.3310/nihropenres.14657.r31006

**Published:** 2024-03-25

**Authors:** Adrian Slee

**Affiliations:** 1 Division of Medicine, University College London, London, England, UK

This is an excellent proposed study protocol which in my opinion follows all key aspects of a well considered study.

- The Abstract is well written with clear aim.
- A decent Introduction to set the scene is included with ESPEN guidelines etc.
- Eligibility criteria is well considered here.
- The assessment data collection section is also well considered. The only aspect I would include alongside would be body composition assessment. 
- The intervention is well thought out and along key guidelines for protein intake which is good to see along with exercise guidelines. This has been well thought out too and good to see the group format with discussion topics too included.
- You have considered key issues such as treatment fidelity and use in intervention logs, adherence,etc.
- You have considered key PPI aspects which are essential in protocol development and it appears you have regular meeting updates too which is good to see.

Well done.

 ps. apologies for the delay in my review

Is the study design appropriate for the research question?

Yes

Is the rationale for, and objectives of, the study clearly described?

Yes

Are sufficient details of the methods provided to allow replication by others?

Yes

Are the datasets clearly presented in a useable and accessible format?

Yes

Reviewer Expertise:

malnutrition, cachexia, sarcopenia, and frailty

I confirm that I have read this submission and believe that I have an appropriate level of expertise to confirm that it is of an acceptable scientific standard.

# Reviewer report: Reviewer response for version 1

**DOI:** 10.3310/nihropenres.14657.r31005

**Published:** 2024-03-25

**Authors:** Konstantinos Prokopidis

**Affiliations:** 1 Department of Musculoskeletal Ageing and Science, University of Liverpool, Liverpool, England, UK

This is an important research, for which, its application may confer beneficial outcomes pre-frail/frail older adults, enhancing their protein intake.

 Some primary comments:

- In the Introduction, a brief description of how frailty is diagnosed in clinical practice may also help your justification of using Fried's criteria.
- In the "Aim" section, you wrote: "...older adults with low protein intake compared to training alone." Did you mean with "...with low protein intake compared to normal protein intake"? This is how it is presented eventually. Sorry for the confusion.
- Table 2, page 7: Are you eventually using only Fried's criteria or multiple questionnaires?

General comments:

- Is there a particular reason you didn't go for 3-day food diaries?
- Have you thought of doing another analysis on observing any differences even between pre-frail vs. frail?
- It would be interesting to see the discrepancies of different age groups (60-69 vs. 70-79 etc.) in using the Good Boost app.
- Regarding the supplement, do you think the added vitamin D and calcium could promote any further "gains" compared to an exclusively protein supplement?

Is the study design appropriate for the research question?

Yes

Is the rationale for, and objectives of, the study clearly described?

Yes

Are sufficient details of the methods provided to allow replication by others?

Yes

Are the datasets clearly presented in a useable and accessible format?

Not applicable

Reviewer Expertise:

Sarcopenia, frailty, malnutrition

I confirm that I have read this submission and believe that I have an appropriate level of expertise to confirm that it is of an acceptable scientific standard, however I have significant reservations, as outlined above.

# Response

**Published:** 2024-04-02

**Authors:** Esther Williamson

**Affiliations:** NDORMS, University of Oxford, Oxford, England, UK

Thank you for taking the time to review our paper and identifying areas for improvement.

 Reviewer feedback:

*1. In the Introduction, a brief description of how frailty is diagnosed in clinical practice may also help your justification of using Fried's criteria.*

**Response:** We have added a sentence to the introduction.

*2. In the "Aim" section, you wrote: "...older adults with low protein intake compared to training alone." Did you mean with "...with low protein intake compared to normal protein intake"? This is how it is presented eventually. Sorry for the confusion.*

**Response:** No, all participants must have low protein intake. I have reworded for clarity.

*3. Table 2, page 7: Are you eventually using only Fried's criteria or multiple questionnaires?*

**Response:** The inclusion criteria are based on the Fried Criteria, but we are collecting other questionnaires used to measure frailty in this feasibility study.

*4. Is there a particular reason you didn't go for 3-day food diaries?*

**Response:** Yes, our patient advisory group were worried about the burden on potential participants which may discourage people from undergoing screening for the study. After discussion with our patient representatives and the study dietitian we reduced this to 2 x 24 hour food diaries making sure that we did one on a week day and one on the weekend to capture variation in diet.

*5. Have you thought of doing another analysis on observing any differences even between pre-frail vs. frail?*

**Response:** As this is a feasibility study with a small sample size, we will not be doing any subgroup analyses of clinical outcomes. However, if we proceed to a fully powered trial, this would be a pre-specified subgroup analyses.

*6. It would be interesting to see the discrepancies of different age groups (60-69 vs. 70-79 etc.) in using the Good Boost app.*

**Response:** Agree, we will look at differences in uptake between older and younger participants.

*7. Regarding the supplement, do you think the added vitamin D and calcium could promote any further "gains" compared to an exclusively protein supplement?*

**Response:** We have used a protein supplement that is specifically formulated for sarcopenia which includes Vitamin D and calcium. Vitamin D is often recommended for people with sarcopenia so may contribute to further gains beyond that of increasing protein alone. We chose this powered supplement as it was less calorific than other available supplements and the main target was increasing protein rather than calories.
